# Use of Casirivimab and Imdevimab for the Treatment of COVID-19

**DOI:** 10.7759/cureus.27766

**Published:** 2022-08-08

**Authors:** Ryan Liu, Rohan Mangal, Thor S Stead, Andrew R Barbera, Latha Ganti

**Affiliations:** 1 Biology, Cedar Park High School, Cedar Park, USA; 2 Medicine, University of Miami Miller School of Medicine, Miami, USA; 3 Medicine, Warren Alpert Medical School of Brown University, Providence, USA; 4 Emergency Medicine, Lakeland Regional Health, Lakeland, USA; 5 Emergency Medicine, HCA Florida Ocala Hospital, Ocala, USA; 6 Emergency Medicine, Envision Physician Services, Plantation, USA; 7 Emergency Medicine, University of Central Florida College of Medicine, Orlando, USA

**Keywords:** imdevimab, casirivimab, regen-cov, casirivimab and imdevimab, monoclonal antibody treatment for covid-19, covid-19, case report

## Abstract

The authors present three cases of unvaccinated coronavirus disease 2019 (COVID-19) patients who exhibited symptoms of fever, sore throat, nausea, diarrhea, congestion, and headache. Although they refused COVID-19 vaccination, they presented for the casirivimab and imdevimab monoclonal antibody cocktail, which resulted in the resolution of all symptoms. The authors describe the mechanisms and importance of monoclonal antibody treatment for high-risk and unvaccinated patients infected with SARS-CoV-2.

## Introduction

In March 2020, the coronavirus disease 2019 (COVID-19) was pronounced a worldwide pandemic [1]. Despite the free cost and wide availability of COVID-19 vaccines to the general public, the United States continues to see an increase in the number of COVID-19 infections due to vaccine hesitancy [2,3]. Most COVID-19 deaths in the United States occur among unvaccinated individuals [4]. Monoclonal antibodies (mAbs) are an important adjunct in the management of COVID-19. A “cocktail” of the IgG1 mAb casirivimab and imdevimab, REGEN-COV, has been shown to significantly decrease hospitalization and mortality in individuals infected with COVID-19 [5]. This treatment has also been shown to decrease the viral load of infected patients and abbreviate the duration of symptoms [6]. REGEN-COV binds noncompeting epitopes of the SARS-CoV-2 spike protein receptor-binding domains, thereby preventing the virus from entering the host cell through the angiotensin-converting enzyme 2 (ACE2) receptor [7]. The authors present three cases of unvaccinated individuals who contracted the Delta variant of the coronavirus and were treated with REGEN-COV. Of note, as of January 24, 2022, the U.S. Food and Drug Administration (FDA) amended the Emergency Use Authorization (EUA) for REGEN-COV to exclude its use in the United States based on Omicron (B.1.1.529) variant being the dominant strain and not susceptible to the treatment. Thus, REGEN-COV remains an investigational drug only as of this writing [8].

## Case presentation

Patient 1 (husband)

**Figure 1:**
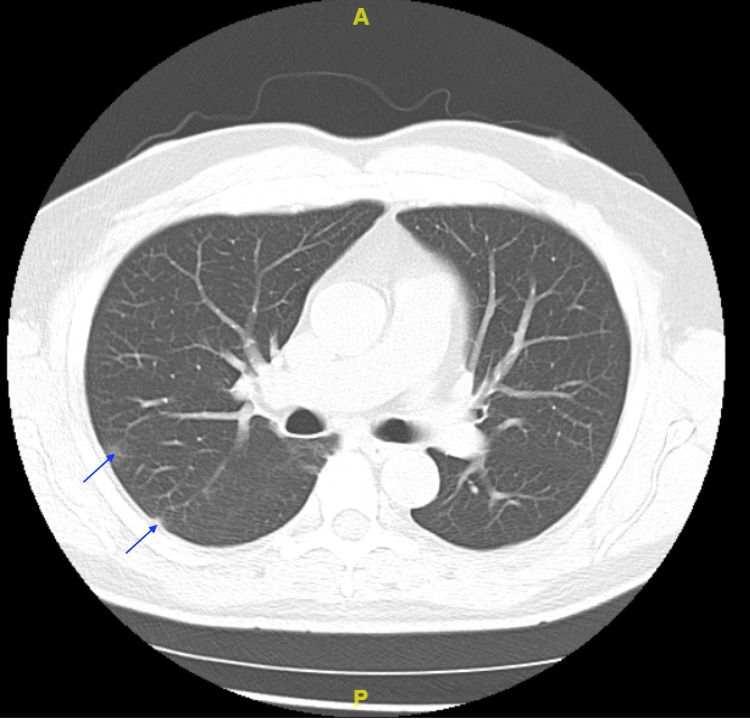
CT scan of the chest (axial view) demonstrating subtle ground-glass opacities (arrows)

A 60-year-old Hispanic male presented to the emergency department (ED) with fever, sore throat, nausea, diarrhea, productive cough, runny nose, and congestion. He denied any headache, loss of taste, loss of smell, chest pain, or shortness of breath. His past medical history was significant for asthma, diabetes, and hypertension. He had no known drug allergies and denied smoking. He had refused the COVID-19 vaccine and had tested positive for COVID-19. On physical examination, his vital signs were as follows: 99.6°F for temperature, respiratory rate of 18 breaths per minute, oxygen saturation of 98% on room air, with a pulse of 74 beats per minute, and blood pressure of 147/82 mmHg. Rhonchi were noted on pulmonary examination. A cardiac examination revealed regular rate and rhythm without murmurs, rubs, or gallops. The remainder of the physical examination was unremarkable. The patient came requesting the mAb infusion and received it. His initial laboratory findings are presented in Table 1. The patient had a chest CT demonstrating areas of increased peripheral “ground-glass” opacities, characteristic of COVID-19 infection (Figure 1). All three patients received chest CT scans, but scans of the mother-in-law and wife were negative for ground-glass opacities.

**Table 1:** Summary of laboratory findings for patients 1, 2, and 3

Laboratory investigation	Patient 1 (husband)	Patient 2 (wife)	Patient 3 (mother)	Reference range
Sodium	137	139	133	135-145 mmol/L
Potassium	4.6	4.1	4.3	3.5-5.3 mmol/L
Chloride	96	99	96	98-107 mmol/L
Carbon dioxide	29	30	25	21-32 mmol/L
Anion gap	12	10	12	4-12 mmol/L
Blood urea nitrogen	19	15	21	7-18 mg/dL
Creatinine	0.9	0.7	1.1	0.6-1.3 mg/dL
Estimated glomerular filtration rate	>60	>60	47	>60
Glucose	162	102	170	74-106 mg/dL
Calcium	9.3	10.6	8.9	8.4-10.2 mg/dL
Total bilirubin	0.5	0.3	0.6	0.0-1.0 mg/dL
Aspartate aminotransferase	26	59	39	15-37 units/L
Alanine aminotransferase	19	70	26	12-78 units/L
Total alkaline phosphatase	70	113	88	45-117 units/L
Lactate dehydrogenase	196	190	253	100-240 units/L
C-reactive protein	1.17	< 0.290	0.351	0-0.300 mg/dL
Total protein	8.1	8.6	7.7	6.4-8.2 g/dL
Albumin	4.8	4.9	4.2	3.4-5.0 g/dL
Coagulation D-dimer	433	320	280	0-500 ng/mL FEU
White blood cell count	5.7	7.9	4.9	4.1-8.3 K/mm^3^
Red blood cell count	4.84	5.15	4.21	3.28-5.50 M/mm^3^
Hemoglobin	15.3	14.1	11.6	12.1-15.1 gm/dL
Hematocrit	45.6	42.3	36.5	35.5-46.9 %
Platelet count	198	270	241	150-450 K/mm^3^
COVID-19 test	Positive	Positive	Positive	Negative

The patient completed the REGEN-COV mAb infusion without experiencing any side effects from the mAb infusion. His saturation leveled out to 95% after the infusion. All symptoms were resolved on the second day after the mAb infusion.

Patient 2 (wife)

A 57-year-old Hispanic female presented to the ED with no symptoms but stated she needed the mAb because her husband was positive for COVID-19. Like him, she too had refused COVID-19 vaccination. She denied any fever, chills, nausea, vomiting, diarrhea, chest pain, shortness of breath, cough, runny nose, congestion, sore throat, loss of taste, or loss of smell. Her past medical history was significant for diabetes, hypertension, Hashimoto’s thyroiditis, seizures, fibromyalgia, and neuropathy. She had known drug allergies to sulfonamide antibiotics and metoclopramide. She denied smoking. She tested positive for COVID-19 in the ED. On physical examination, her temperature was 98.5°F, respiratory rate was 18 breaths per minute, oxygen saturation was 98% on room air, pulse rate was 70 beats per minute, and blood pressure was 167/87 mmHg. Pulmonary examination revealed lungs clear to auscultation, without rales, or rhonchi. A cardiac examination revealed a regular rate and rhythm without murmurs, rubs, or gallops. The remainder of her physical examination was unremarkable. The patient completed the REGEN-COV mAb infusion and did not experience any side effects.

Patient 3 (mother of patient 1)

An 80-year-old Hispanic female presented to the ED with fever, headache, diarrhea, and dry cough of four-day duration. She denied chest pain, shortness of breath, nausea, vomiting, or runny nose. Her past medical history was significant for diabetes and hypertension. Her drug allergies included penicillins. She denied smoking. She too had refused the COVID-19 vaccine, like her son and daughter-in-law. She too tested positive for COVID-19 in the ED. On physical examination, her temperature was 98.5°F, respiratory rate was 18 breaths per minute, oxygen saturation was 93% on room air, pulse rate was 84 beats per minute, and blood pressure was 143/69 mmHg. Pulmonary examination revealed lungs clear to auscultation, and no rales or rhonchi were noted. A cardiac examination revealed a regular rate and rhythm without murmurs, rubs, or gallops. The remainder of her physical examination was unremarkable. Her initial laboratory findings are presented in Table 1. The patient completed the REGEN-COV mAb infusion and did not experience any side effects.

In December 2021, the Omicron variant became dominant in the United States. Sotrovimab was shown to be the only effective mAb against the Omicron variant. None of the patients received the sotrovimab mAb infusion.

## Discussion

**Figure 2:**
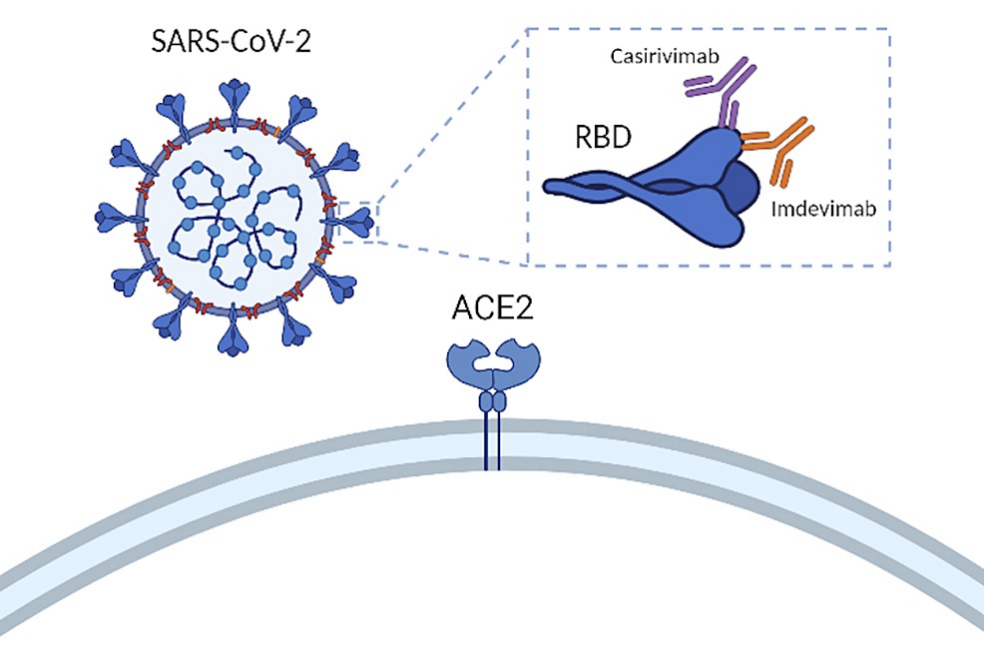
Inhibition of SARS-CoV-2 host cell engagement by REGEN-COV Figure designed by Ryan Liu and created on biorender.com. RBD: receptor-binding domains; ACE2: angiotensin-converting enzyme 2.

With the recent wave of pre- and post-exposure individuals wanting to receive neutralizing mAbs, it is essential to distinguish the populations for which mAbs are currently appropriate. Individuals whose immediate protection is at-risk and individuals who can increase the spread and transmissibility of SARS-CoV-2 infection are the target population for REGEN-COV. REGEN-COV is especially effective in enhancing the clearance of the COVID-19 virus in patients whose immune response has not yet been initiated or in patients with a high baseline viral load. The EUA for neutralizing mAbs is for use in non-hospitalized patients 12 years or older, weighing more than 40 kg, with mild or moderate symptoms, and with one or more risk factors for progression to severe disease. These risk factors include obesity (BMI ≥ 25 kg/m^2^), old age (age ≥ 65 years), pregnancy, diabetes, kidney disease, reduced immune function, cardiovascular disease, lung disease, developmental disorders, and sickle cell disease [9]. An advantage of REGEN-COV over other COVID-19 treatments is that they have considerable mutational tolerance of receptor-binding domains (RBD). RNA viruses, such as SARS-CoV-2, can rapidly evolve and develop resistance to specific therapeutics. At the beginning of the COVID-19 pandemic, only a small amount of sequence divergence was observed. This slow divergence is likely due to the coronavirus exonuclease “proofreading” activity that scanned for any replication errors. This caused scientists to underestimate the virus’ risk for resistance to vaccination and mAb administration. However, the selection of resistant variants from RBD mAbs provides evidence of a high degree of plasticity in this region [10]. Because of this, the potential for treatment-induced escape mutants is a significant problem for anti-COVID-19 therapeutics. The initial solution for this issue was to select mAbs that correspond to epitopes that retain multiple strains of the same virus. However, this strategy may not suffice. “Cocktail” therapies, as shown in Figure 2, in which two antibodies are selected to bind to distinct regions of the viral target are a potential solution to minimize mutational escape by SARS-CoV-2 [11].

From a clinical standpoint, adverse effects of mAbs include infusion-related reactions. These are characterized by flushing, fever, chills, nausea, vomiting, back pain, abdominal pain, pruritus, or skin rashes. These symptoms typically present 30 to 60 minutes after initiating the infusion. Most infusion-related reactions disappear when the infusion is stopped. Although evidence of natural immunity in unvaccinated individuals was present up to 20 months after the COVID-19 infection [12], the most crucial step to contain the COVID-19 global pandemic is vaccinations to prevent SARS-CoV-2 infections from spreading in communities, to begin with [13].

## Conclusions

This case series describes three unvaccinated COVID-19 patients treated with casirivimab and imdevimab mAbs early in the disease course, resulting in prompt resolution of their symptoms. Unvaccinated individuals are most at risk for contracting and spreading the SARS-CoV-2 infection. Although mAb is an effective treatment in patients with risk factors for progression to severe COVID-19, increasing vaccination rates is the cornerstone of controlling COVID-19 spread.
